# Survival analysis in patients with invasive lobular cancer and invasive ductal cancer according to hormone receptor expression status in the Korean population

**DOI:** 10.1371/journal.pone.0262709

**Published:** 2022-01-20

**Authors:** Douk Kwon, Byung Kyun Ko, Seung Pil Jung, Hong-Kyu Kim, Eun-Kyu Kim, Yong Sik Jung, Hyun Jo Youn, Sae Byul Lee

**Affiliations:** 1 Department of Surgery, University of Ulsan College of Medicine, Asan Medical Center, Seoul, Korea; 2 Department of General Surgery, College of Medicine, Ulsan University Hospital, Ulsan, Korea; 3 Division of Breast and Endocrine Surgery, Department of Surgery, Korea University Anam Hospital, Korea University College of Medicine, Seoul, Korea; 4 Department of Surgery, Breast Care Center, Seoul National University Hospital, Seongnam, Korea; 5 Department of Surgery, Seoul National University College of Medicine, Breast Care Center, Seoul National University Bundang Hospital, Seongnam, Korea; 6 Department of Surgery, Breast Cancer Center, Ajou University School of Medicine, Suwon, Korea; 7 Department of Surgery, Jeonbuk National University Medical School, Jeonju, Korea; Nine of July University (UNINOVE): Discipline of Medical Oncology - Post Graduation Program in Medicine, BRAZIL

## Abstract

**Background:**

We compared the clinicopathological characteristics and survival outcomes of invasive lobular carcinoma (ILC) cases with those of invasive ductal carcinoma (IDC) cases in various hormone receptor expression subgroups.

**Methods:**

We compared clinicopathological characteristics, overall survival (OS), and breast cancer-specific survival (BCSS) between patients with IDC (n = 95,486) and ILC (n = 3,023). In addition, we analyzed the effects of different hormone receptor expression subgroups on survival.

**Results:**

The ILC group had more instances of advanced stage and hormonal receptor positivity than did the IDC group (p < 0.001), but the IDC group had higher histological grade and nuclear grade, as well as higher frequency of human epidermal growth factor receptor 2 and Ki67 expression than did the ILC group (p < 0.001). The OS and BCSS were not significantly different between the IDC and ILC groups. The 5-year OS of the IDC group was 88.8%, while that of the ILC group was 90.6% (p = 0.113). The 5-year BCSS of the IDC group was 94.8%, while that of the ILC group was 95.0% (p = 0.552). When analyzing each hormone receptor expression subgroup, there were no significant differences in survival between the IDC and ILC groups. However, the estrogen receptor (ER) negative/progesterone receptor (PR) negative subgroup showed differences in survival between the IDC and ILC groups. Moreover, the hazard ratio of ILC in the ER negative/PR negative subgroup was 1.345 (95% confidence interval: 1.012–1.788; p = 0.041).

**Conclusions:**

Hormone receptor expression should be considered when determining prognosis and treatment regimen for IDC and ILC. Researchers should further study the ER negative/PR negative population to identify treatment and prognostic models that will facilitate the development of individualized therapy for these patients, which is needed for good outcomes.

## Introduction

In Korea, just as in western countries, the prevalence of breast cancer is increasing [1–4], and invasive breast cancer accounts for most cases [5]. Several studies comparing invasive ductal carcinoma (IDC) and invasive lobular carcinoma (ILC) have been conducted worldwide [6–10]; these have shown that ILC cases have similar or better survival outcomes compared to those of IDC cases, which account for most invasive breast cancer cases [8–12].

As individualized therapy has become important, studies on hormone receptor expression subtypes have been conducted, mainly in the West. According to the Surveillance, Epidemiology, and End Results (SEER) Program database, compared to IDC, ILC is associated with larger tumor size, older diagnosis age, advanced stage, lower histological grade, higher estrogen receptor (ER)/progesterone receptor (PR) expression, and lower human epidermal growth factor receptor 2 (HER-2) expression. Higher percentages of lymph node positivity and distant metastasis are also found in ILC cases than in IDC cases. In an analysis of hormone receptor expression status that excluded the ER negative/PR negative subgroup, the ER positive/PR positive subgroup showed the best survival, while the ER positive/PR negative subgroup had the worst outcomes [6].

As in the West, studies are being conducted in Asia, including Korea [13–15]. However, few have compared invasive breast cancer survival outcomes among different hormone receptor expression subgroups. Therefore, in the present work, we conducted a study on invasive breast cancer in Korea using data from the Korean Breast Cancer Registry (KBCR) to compare and analyze survival among various hormone receptor expression subgroups.

## Materials and methods

### Ethical approval

All procedures performed in studies involving human participants were in accordance with the ethical standards of the institutional and/or national research committee and with the 1964 Helsinki Declaration and its later amendments.

### Patient selection

In the KBCR database, we identified 98,509 patients with invasive breast cancer diagnosed between 2001 and 2013 who aged more than 18 years old. The KBCR database is a nationwide, Korean, multi-institutional online database. The Korean Breast Cancer Society (KBCS) prospectively keeps the information of patients diagnosed with breast cancer in 102 hospitals. The following information is included: patient identification number, age at operation, sex, tumor stage based on the American Joint Committee on Cancer classification, pathophysiology, and type of surgery. Expression of ER and PR was considered positive if more than 10% of the tumor stained positive, HER-2 status was evaluated using HER-2 overexpression analysis with any grade over 2+ being considered positive. Fluorescence *in situ* hybridization was used when HER-2 status was graded as 2+, and considered positive if graded 3+ for its result. We excluded patients with metastatic breast cancer at the time of diagnosis, as well as those with carcinoma *in situ* or poorly evaluated axillary lymph nodes, and those without biological subtype information [S1 Table]. This study was approved by the Institutional Review Board of Asan Medical Center, Seoul, South Korea (20171341). Given that the study was based on retrospective clinical data, the need for informed consent was waived.

### Statistical analysis

The clinicopathological features of invasive breast cancer cases were analyzed using a Pearson’s chi-square test. We used the Kaplan–Meier method and log-rank test to analyze and compare survival outcomes. Overall survival (OS) was defined as the time from the date of breast cancer diagnosis until the date of death (from any cause) or last follow-up. Breast cancer-specific survival (BCSS) was defined as the time from the date of breast cancer diagnosis until the date of breast cancer-related death or last follow-up. A Cox proportional hazard analysis was used to obtain hazard ratios (HRs) with 95% confidence intervals (CIs) in uni- and multivariable analyses. All p-values less than 0.05 were considered statistically significant. We used SPSS statistical software, version 26.0 (SPSS Inc., Chicago, USA) for all statistical analysis.

## Results

### Clinicopathological characteristics of patients with invasive breast cancer

In total, 98,509 patients diagnosed with invasive breast cancer between 2001 and 2013 were selected from the KBCR database, and their data were analyzed. Among them, 95,486 (96.9%) patients had IDC and 3,023 (3.1%) had ILC. The clinicopathological characteristics of the study population are summarized in Table 1. Patients with ILC were older at the time of surgery than those with IDC (≥41 years of age at operation: ILC group, 89.3% vs. IDC group, 81.5%; p < 0.001). Compared to the IDC group, the ILC group more frequently presented with advanced stage and positive ER and PR expression (p < 0.001). The IDC group had higher histological grade and nuclear grade as well as higher frequency of HER-2 and Ki67 expression than did the ILC group (p < 0.001).

**Table 1:** Clinicopathological characteristics of patients with invasive breast cancer.

Characteristics	Total (n = 98509)	IDC group (n = 95486)	ILC group (n = 3023)	p-value
Age at operation				
≤ 40	17998 (18.3)	17674 (18.5)	324 (10.7)	< 0.001
≥ 41	80511 (81.7)	77812 (81.5)	2699 (89.3)	
T stage				
0	115 (0.1)	112 (0.1)	3 (0.1)	< 0.001
1	55219 (56.1)	53756 (56.3)	1463 (48.4)	
2	38045 (38.6)	36786 (38.5)	1259 (41.6)	
3	4212 (4.3)	3935 (4.1)	277 (9.2)	
4	918 (0.9)	897 (0.9)	21 (0.7)	
N stage				
0	63061 (64.0)	61099 (64.0)	1962 (64.9)	0.018
1	25387 (25.8)	24664 (25.8)	723 (23.9)	
2	6824 (6.9)	6608 (6.9)	216 (7.1)	
3	3237 (3.3)	3115 (3.3)	122 (4.0)	
TNM stage				
I	42209 (42.9)	41032 (43.0)	1177 (38.9)	< 0.001
II	44492 (45.2)	43090 (45.1)	1402 (46.4)	
III	11786 (12.0)	11342 (11.9)	444 (14.7)	
Unknown	22	22	0	
Histologic grade				
G1	14880 (17.4)	14411 (17.3)	469 (24.7)	< 0.001
G2	39257 (46.0)	38079 (45.7)	1178 (62.1)	
G3	31163 (36.5)	30914 (37.1)	249 (13.1)	
Unknown	13209	12082	1127	
Nuclear grade				
G1	8675 (11.7)	8211 (11.3)	464 (24.4)	< 0.001
G2	36776 (49.5)	35597 (49.1)	1179 (62.0)	
G3	28909 (38.9)	28649 (39.5)	260 (13.7)	
Unknown	24149	23029	1120	
LVI				
Negative	54057 (68.6)	51991 (68.1)	2066 (82.2)	< 0.001
Positive	24768 (31.4)	24321 (31.9)	447 (17.8)	
Unknown	19684	19174	510	
Hormone expression				
ER+/PR+	50367 (54.1)	48211 (53.4)	2156 (73.8)	< 0.001
ER+/PR−	11078 (11.9)	10660 (11.8)	418 (14.3)	
ER−/PR+	4372 (4.7)	4270 (4.7)	102 (3.5)	
ER−/PR−	27327 (29.3)	27082 (30.0)	245 (8.4)	
Unknown	5365	5263	102	
HER2				
Negative	67276 (79.4)	64753 (78.9)	2523 (93.9)	< 0.001
Positive	17503 (20.6)	17338 (21.1)	165 (6.1)	
Unknown	13730	13395	335	
Ki67				
≤ 20	26933 (62.4)	25713 (61.6)	1220 (85.7)	< 0.001
> 20	16247 (37.6)	16044 (38.4)	203 (14.3)	
Unknown	55329	53729	1600	
Chemotherapy				
No	22732 (25.9)	21930 (25.8)	802 (29.1)	< 0.001
Yes	65033 (74.1)	63082 (74.2)	1951 (70.9)	
Unknown	10744	10474	270	
Radiation therapy				
No	32528 (38.7)	31461 (38.7)	1067 (40.1)	0.067
Yes	51486 (61.3)	49894 (61.3)	1592 (59.9)	
Unknown	14495	14131	364	
Hormonal therapy				
No	24402 (29.8)	24098 (30.4)	304 (11.6)	< 0.001
Yes	57460 (70.2)	55154 (69.6)	2306 (88.4)	
Unknown	16647	16234	413	
Surgery				
TM	47951 (49.4)	46337 (49.2)	1614 (54.0)	< 0.001
BCS	49203 (49.4)	47828 (50.8)	1375 (46.0)	
Unknown	1355	1321	34	
Axillary op				
SNB	25320 (26.0)	24328 (25.8)	992 (33.1)	< 0.001
ALND	67578 (69.4)	65711 (69.6)	1867 (62.3)	
No op	4508 (4.6)	4372 (4.6)	136 (4.5)	
Unknown	1103	1075	28	

ALND, axillary lymph node dissection; BCS, breast conserving surgery; ER, estrogen receptor; HER2, human epidermal growth factor receptor 2; IDC, invasive ductal carcinoma; ILC, invasive lobular carcinoma; LVI, lymphovascular invasion; PR, progesterone receptor; SNB, sentinel node biopsy; TM, total mastectomy.

In Table 2, we have compared the clinicopathological characteristics of the study population according to the hormonal receptor expression subgroups. The ER negative (−)/PR− group presented much higher histological grade and nuclear grade, as well as higher frequency of HER-2 and Ki67 expression than did other groups (p < 0.001). Regarding TNM stage, the ER positive (+)/PR+ subgroup was the least advanced, while the ER−/PR+ subgroup was the most advanced (p < 0.001). Patients in the ER−/PR+ were the oldest and showed the highest frequency of lymphovascular invasion (p < 0.001).

**Table 2:** Clinicopathological characteristics of hormonal expression subgroups in the total study population.

	Total population				
Characteristics	ER+/PR+(n = 50367)	ER+/PR−(n = 11078)	ER−/PR+(n = 4372)	ER−/PR−(n = 27327)	p-value
Age at operation					
≤ 40	8722 (17.3)	1565 (14.1)	979 (22.4)	5390 (19.7)	< 0.001
≥ 41	41645 (82.7)	9513 (85.9)	3393 (77.6)	21937 (80.2)	
T stage					
0	27 (0.1)	21 (0.2)	2 (0.0)	59 (0.2)	< 0.001
1	31135 (61.8)	6323 (57.1)	1972 (45.1)	13326 (48.8)	
2	17223 (34.2)	4115 (37.1)	2063 (47.2)	12199 (44.6)	
3	1688 (3.4)	484 (4.4)	280 (6.4)	1398 (5.1)	
4	294 (0.6)	135 (1.2)	55 (1.3)	345 (1.3)	
N stage					
0	32345 (64.2)	6877 (62.1)	2556 (58.5)	17858 (65.3)	< 0.001
1	13392 (26.6)	2979 (26.9)	1241 (28.4)	6347 (23.2)	
2	3168 (6.3)	862 (7.8)	399 (9.1)	1993 (7.3)	
3	1462 (2.9)	360 (3.2)	176 (4.0)	1129 (4.1)	
TNM stage					
I	23693 (47.0)	4752 (42.9)	1457 (33.3)	10414 (38.1)	< 0.001
II	21412 (42.5)	4859 (43.9)	2220 (50.8)	13230 (48.4)	
III	5261 (10.4)	1463 (13.2)	694 (15.9)	3677 (13.5)	
Unknown	1	4	1	6	
Histologic grade					
G1	11285 (24.9)	1652 (16.7)	444 (12.3)	971 (4.1)	< 0.001
G2	24339 (53.7)	5140 (52.0)	1503 (41.6)	7006 (29.3)	
G3	9716 (21.4)	3094 (31.3)	1662 (46.1)	15957 (66.7)	
Unknown	5027	1192	763	3393	
Nuclear grade					
G1	5846 (14.5)	1012 (11.8)	330 (11.5)	1180 (5.6)	< 0.001
G2	24565 (61.1)	4591 (53.4)	1220 (42.5)	5493 (26.1)	
G3	9768 (24.3)	2996 (34.8)	1323 (46.0)	14346 (68.3)	
Unknown	10188	2479	1499	6308	
LVI					
Negative	29567 (68.9)	6258 (68.9)	1898 (61.6)	15324 (69.6)	< 0.001
Positive	13344 (31.1)	2822 (31.1)	1182 (38.4)	6692 (30.4)	
Unknown	7456	1998	1292	5311	
HER2					
Negative	40713 (87.7)	7808 (78.0)	2674 (73.9)	15868 (64.9)	< 0.001
Positive	5712 (12.3)	2198 (22.0)	945 (26.1)	8589 (35.1)	
Unknown	3942	1072	753	2870	
Ki67					
≤ 20	18369 (74.2)	3158 (68.0)	694 (51.4)	4624 (37.6)	< 0.001
> 20	6389 (25.8)	1485 (32.0)	656 (48.6)	7684 (62.4)	
Unknown	25609	6435	3022	15019	
Chemotherapy					
No	15274 (33.3)	2991 (30.3)	602 (15.6)	3109 (12.5)	< 0.001
Yes	30561 (66.7)	6887 (69.7)	3261 (84.4)	21735 (87.5)	
Unknown	4532	1200	509	2483	
Radiation therapy					
No	15693 (35.3)	3883 (40.7)	1567 (43.7)	9700 (41.1)	< 0.001
Yes	28707 (64.7)	5653 (59.3)	2019 (56.3)	13910 (58.9)	
Unknown	5967	1542	786	3717	
Hormonal therapy					
No	2545 (5.8)	796 (8.5)	521 (15.1)	19473 (86.9)	< 0.001
Yes	41310 (94.2)	8624 (91.5)	2927 (84.9)	2937 (13.1)	
Unknown	6512	1658	924	4917	
Surgery					
TM	21685 (43.6)	5710 (52.2)	2456 (57.2)	14392 (53.3)	< 0.001
BCS	28054 (56.4)	5230 (47.8)	1840 (42.8)	12634 (46.7)	
Unknown	628	138	76	301	
Axillary op					
SNB	15399 (30.9)	2783 (25.4)	528 (12.2)	6363 (23.5)	< 0.001
ALND	32146 (64.5)	7703 (70.2)	3576 (82.9)	19469 (71.9)	
No op	2302 (4.6)	486 (4.4)	209 (4.8)	1252 (4.6)	
Unknown	520	106	59	243	

ALND, axillary lymph node dissection; BCS, breast conserving surgery; ER, estrogen receptor; HER2, human epidermal growth factor receptor 2; LVI, lymphovascular invasion; PR, progesterone receptor; SNB, sentinel node biopsy; TM, total mastectomy.

**Table 2–1:** Clinicopathological characteristics of hormonal expression subgroups in the IDC group.

	IDC group				
Characteristics	ER+/PR+(n = 50367)	ER+/PR−(n = 11078)	ER−/PR−(n = 4372)	ER−/PR−(n = 27327)	p-value
Age at operation					
≤ 40	8494 (17.6)	1542 (14.5)	962 (22.5)	5362 (19.8)	< 0.001
≥ 41	39717 (82.4)	9118 (85.5)	3308 (77.5)	21720 (80.2)	
T stage					
0	26 (0.1)	21 (0.2)	2 (< 0.1)	59 (0.2)	< 0.001
1	30069 (62.4)	6119 (57.4)	1930 (45.2)	13227 (48.8)	
2	16330 (33.9)	3957 (37.1)	2012 (47.1)	12084 (44.6)	
3	1502 (3.1)	436 (4.1)	271 (6.3)	1370 (5.1)	
4	284 (0.6)	127 (1.2)	55 (1.3)	342 (1.3)	
N stage					
0	30927 (64.1)	6596 (61.9)	2501 (58.6)	17712 (65.4)	< 0.001
1	12859 (26.7)	2896 (27.2)	1212 (28.4)	6293 (23.2)	
2	3039 (6.3)	825 (7.7)	387 (9.1)	1964 (7.3)	
3	1386 (2.9)	343 (3.2)	170 (4.0)	1113 (4.1)	
TNM stage					
I	22834 (47.4)	4579 (43.0)	1428 (33.5)	10337 (38.2)	< 0.001
II	20390 (42.3)	4688 (44.0)	2167 (50.8)	13119 (48.5)	
III	4986 (10.3)	1389 (13.0)	674 (15.8)	3620 (13.4)	
Unknown	1	4	1	6	
Histologic grade					
G1	10899 (24.8)	1600 (16.6)	433 (12.2)	955 (4.0)	< 0.001
G2	23446 (53.4)	4967 (51.6)	1473 (41.4)	6939 (29.1)	
G3	9555 (21.8)	3063 (31.8)	1652 (46.4)	15912 (66.8)	
Unknown	4311	1030	712	3276	
Nuclear grade					
G1	5470 (14.1)	957 (11.5)	317 (11.2)	1165 (5.6)	< 0.001
G2	23664 (61.1)	4420 (53.0)	1190 (42.1)	5427 (26.0)	
G3	9603 (24.8)	2959 (35.5)	1319 (46.7)	14298 (68.4)	
Unknown	9474	2324	1444	6192	
LVI					
Negative	28007 (68.2)	5969 (68.4)	1838 (61.1)	15188 (69.6)	< 0.001
Positive	13041 (31.8)	2760 (31.6)	1169 (38.9)	6633 (30.4)	
Unknown	7163	1931	1263	5261	
HER2					
Negative	38804 (87.4)	7454 (77.4)	2587 (73.3)	15702 (64.8)	< 0.001
Positive	5618 (12.6)	2174 (22.6)	941 (26.7)	8547 (35.2)	
Unknown	3789	1032	742	2833	
Ki67					
≤ 20	17426 (73.6)	2976 (67.0)	672 (50.7)	4553 (37.3)	< 0.001
> 20	6240 (26.4)	1467 (33.0)	653 (49.3)	7653 (62.7)	
Unknown	24545	6217	2945	14876	
Chemotherapy					
No	14668 (33.5)	2862 (30.1)	585 (15.5)	3077 (12.5)	< 0.001
Yes	29175 (66.5)	6638 (69.9)	3184 (84.5)	21541 (87.5)	
Unknown	4368	1160	501	2464	
Radiation therapy					
No	14918 (35.1)	3746 (40.8)	1531 (43.8)	9606 (41.1)	< 0.001
Yes	27550 (64.9)	5425 (59.2)	1965 (56.2)	13791 (58.9)	
Unknown	5743	1489	774	3685	
Hormonal therapy					
No	2451 (5.8)	761 (8.4)	515 (15.3)	19317 (87.0)	< 0.001
Yes	39500 (94.2)	8297 (91.6)	2851 (84.7)	2887 (13.0)	
Unknown	6260	1602	904	4878	
Surgery					
TM	20590 (43.2)	5482 (52.1)	2392 (57.0)	14235 (53.2)	< 0.001
BCS	27017 (56.8)	5044 (47.9)	1805 (43.0)	12547 (46.8)	
Unknown	604	134	73	300	
Axillary op					
SNB	14612 (30.6)	2651 (25.1)	518 (12.3)	6308 (23.5)	< 0.001
ALND	30889 (64.7)	7444 (70.5)	3491 (82.8)	19288 (71.9)	
No op	2210 (4.6)	462 (4.4)	205 (4.9)	1243 (4.6)	
Unknown	500	103	56	243	

ALND, axillary lymph node dissection; BCS, breast conserving surgery; ER, estrogen receptor; HER2, human epidermal growth factor receptor 2; IDC, invasive ductal carcinoma; LVI, lymphovascular invasion; PR, progesterone receptor; SNB, sentinel node biopsy; TM, total mastectomy.

**Table 2–2:** Clinicopathological characteristics of hormonal expression subgroups in ILC group.

	ILC group				
Characteristics	ER+/PR+(n = 50367)	ER+/PR−(n = 11078)	ER−/PR+(n = 4372)	ER−/PR−(n = 27327)	p-value
Age at operation					
≤ 40	228 (10.6)	23 (5.5)	17 (16.7)	28 (11.4)	0.001
≥ 41	1928 (89.4)	395 (94.5)	85 (83.3)	217 (88.6)	
T stage					
0	1 (< 0.1)	0 (< 0.1)	0 (< 0.1)	0 (< 0.1)	0.010
1	1066 (49.4)	204 (48.8)	42 (41.2)	99 (40.4)	
2	893 (41.4)	158 (37.8)	51 (50.0)	115 (46.9)	
3	186 (8.6)	48 (11.5)	9 (8.8)	28 (11.4)	
4	10 (0.5)	8 (1.9)	0 (< 0.1)	3 (1.2)	
N stage					
0	1418 (65.8)	281 (67.2)	55 (53.9)	146 (59.6)	< 0.001
1	533 (24.7)	83 (19.9)	29 (28.4)	54 (22.0)	
2	129 (6.0)	37 (8.9)	12 (11.8)	29 (11.8)	
3	76 (3.5)	17 (4.1)	6 (5.9)	16 (6.5)	
TNM stage					
I	859 (39.8)	173 (41.4)	29 (28.4)	77 (31.4)	< 0.001
II	1022 (47.4)	171 (40.9)	53 (52.0)	111 (45.3)	
III	275 (12.8)	74 (17.7)	20 (19.6)	57 (23.3)	
Histologic grade					
G1	386 (26.8)	52 (20.3)	11 (21.6)	16 (12.5)	< 0.001
G2	893 (62.0)	173 (67.6)	30 (58.8)	67 (52.3)	
G3	161 (11.2)	31 (12.1)	10 (19.6)	45 (35.2)	
Unknown	716	162	51	117	
Nuclear grade					
G1	376 (26.1)	55 (20.9)	13 (27.7)	15 (11.6)	< 0.001
G2	901 (62.5)	171 (65.0)	30 (63.8)	66 (51.2)	
G3	165 (11.4)	37 (14.1)	4 (8.5)	48 (37.2)	
Unknown	714	155	55	116	
LVI					
Negative	1560 (83.7)	289 (82.3)	60 (82.2)	136 (69.7)	< 0.001
Positive	303 (16.3)	62 (17.7)	13 (17.8)	59 (30.3)	
Unknown	293	67	29	50	
HER2					
Negative	1909 (95.3)	354 (93.7)	87 (95.6)	166 (79.8)	< 0.001
Positive	94 (4.7)	24 (6.3)	4 (4.4)	42 (20.2)	
Unknown	153	40	11	37	
Ki67					
≤ 20	943 (86.4)	182 (91.0)	22 (88.0)	71 (69.6)	< 0.001
> 20	149 (13.6)	18 (9.0)	3 (12.0)	31 (30.4)	
Unknown	1064	218	77	143	
Chemotherapy					
No	606 (30.4)	129 (34.1)	17 (18.1)	32 (14.2)	< 0.001
Yes	1386 (69.6)	249 (65.9)	77 (81.9)	194 (85.8)	
Unknown	164	40	8	19	
Radiation therapy					
No	775 (40.1)	137 (37.5)	36 (40.0)	94 (44.1)	0.486
Yes	1157 (59.9)	228 (62.5)	54 (60.0)	119 (55.9)	
Unknown	224	53	12	32	
Hormonal therapy					
No	94 (4.9)	35 (9.7)	6 (7.3)	156 (75.7)	< 0.001
Yes	1810 (95.1)	327 (90.3)	76 (92.7)	50 (24.3)	
Unknown	252	56	20	39	
Surgery					
TM	1095 (51.4)	228 (55.1)	64 (64.6)	157 (64.3)	< 0.001
BCS	1037 (48.6)	186 (44.9)	35 (35.4)	87 (35.7)	
Unknown	24	4	3	1	
Axillary op					
SNB	787 (36.8)	132 (31.8)	10 (10.1)	55 (22.4)	< 0.001
ALND	1257 (58.8)	259 (62.4)	85 (85.9)	181 (73.9)	
No op	92 (4.3)	24 (5.8)	4 (4.0)	9 (3.7)	
Unknown	20	3	3	0	

ALND, axillary lymph node dissection; BCS, breast conserving surgery; ER, estrogen receptor; HER2, human epidermal growth factor receptor 2; ILC, invasive lobular carcinoma; LVI, lymphovascular invasion; PR, progesterone receptor; SNB, sentinel node biopsy; TM, total mastectomy.

### Comparing survival outcomes of invasive breast cancer

**Fig 1:**
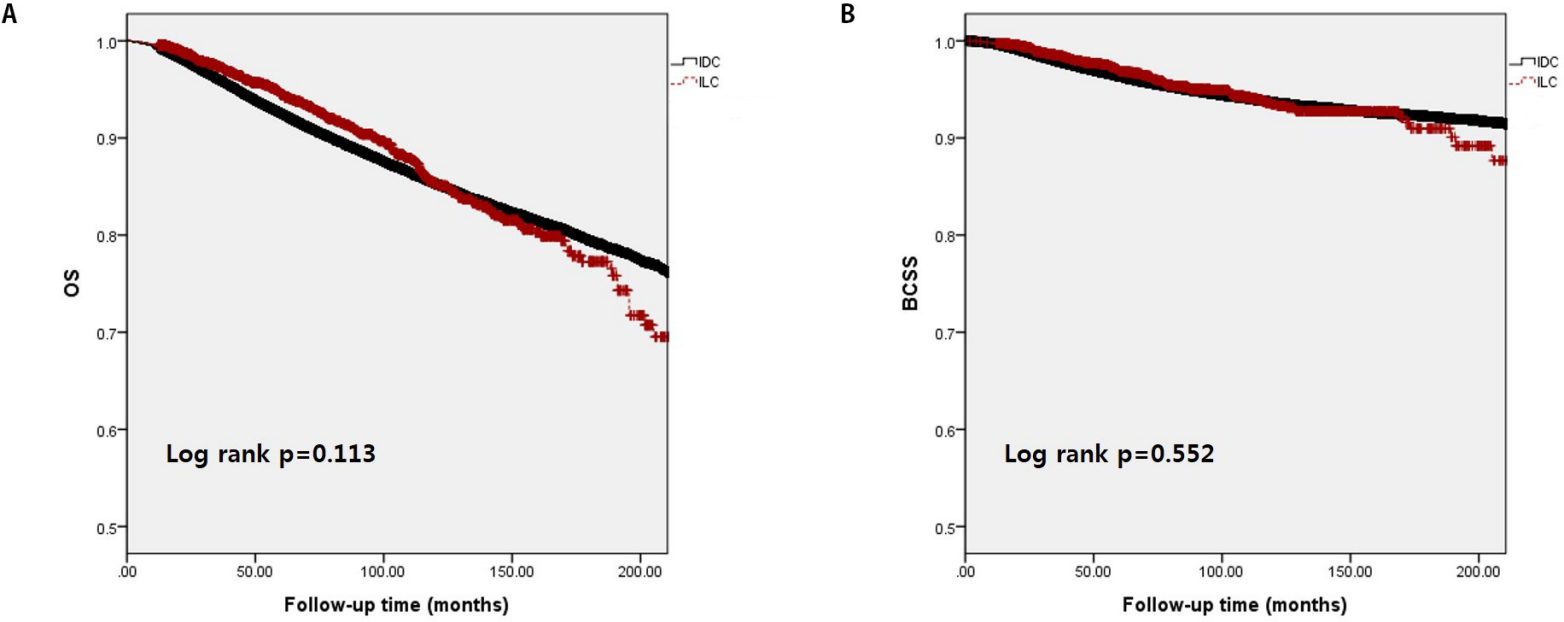
Kaplan–Meier survival analysis of overall survival (OS) (A) and breast cancer-specific survival (BCSS) (B) between the invasive ductal carcinoma and invasive lobular carcinoma groups.

The median follow-up period of the study population was 76.9 months (range: 0.1–304 months). Fig 1 shows no significant differences in survival between the IDC and ILC groups. The 5-year OS of the IDC group was 88.8%, while that of the ILC group was 90.6% (p = 0.113). The 5-year BCSS of the IDC group was 94.8%, while that of the ILC group was 95.0% (p = 0.552).

**Fig 2:**
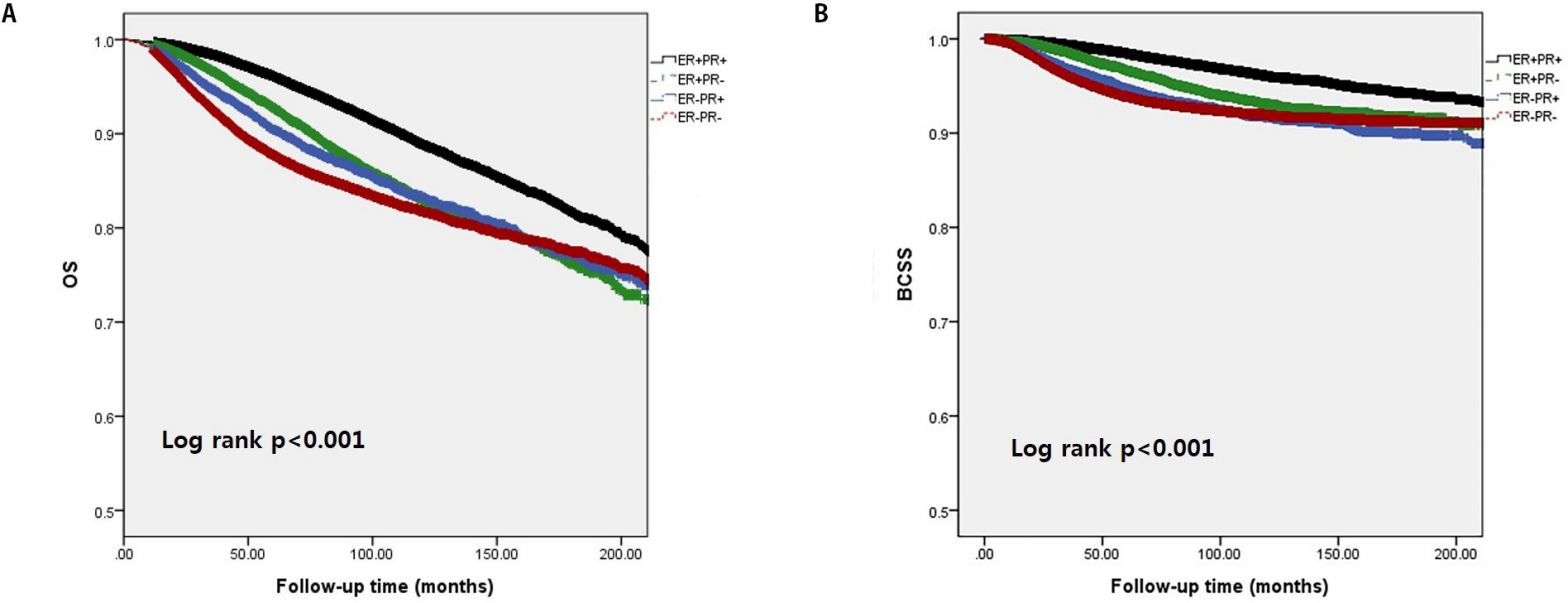
Kaplan–Meier survival analysis of overall survival (OS) (A) and breast cancer-specific survival (BCSS) (B) according to estrogen receptor and progesterone receptor status.

In Fig 2, we analyzed the comparison of 5-year survival outcomes among hormone receptor expression subgroups in the total population. The ER+/PR+ subgroup showed the best 5-year survival (OS: 96.1%, BCSS: 98.5%) followed by the ER+/PR− subgroup (OS: 92.8%, BCSS: 96.7%), ER −/PR+ subgroup (OS: 90.5%, BCSS: 94.7%), and ER−/PR− subgroup (OS: 87.8%, BCSS: 91.7%; p < 0.001 for both OS and BCSS).

**Fig 3:**
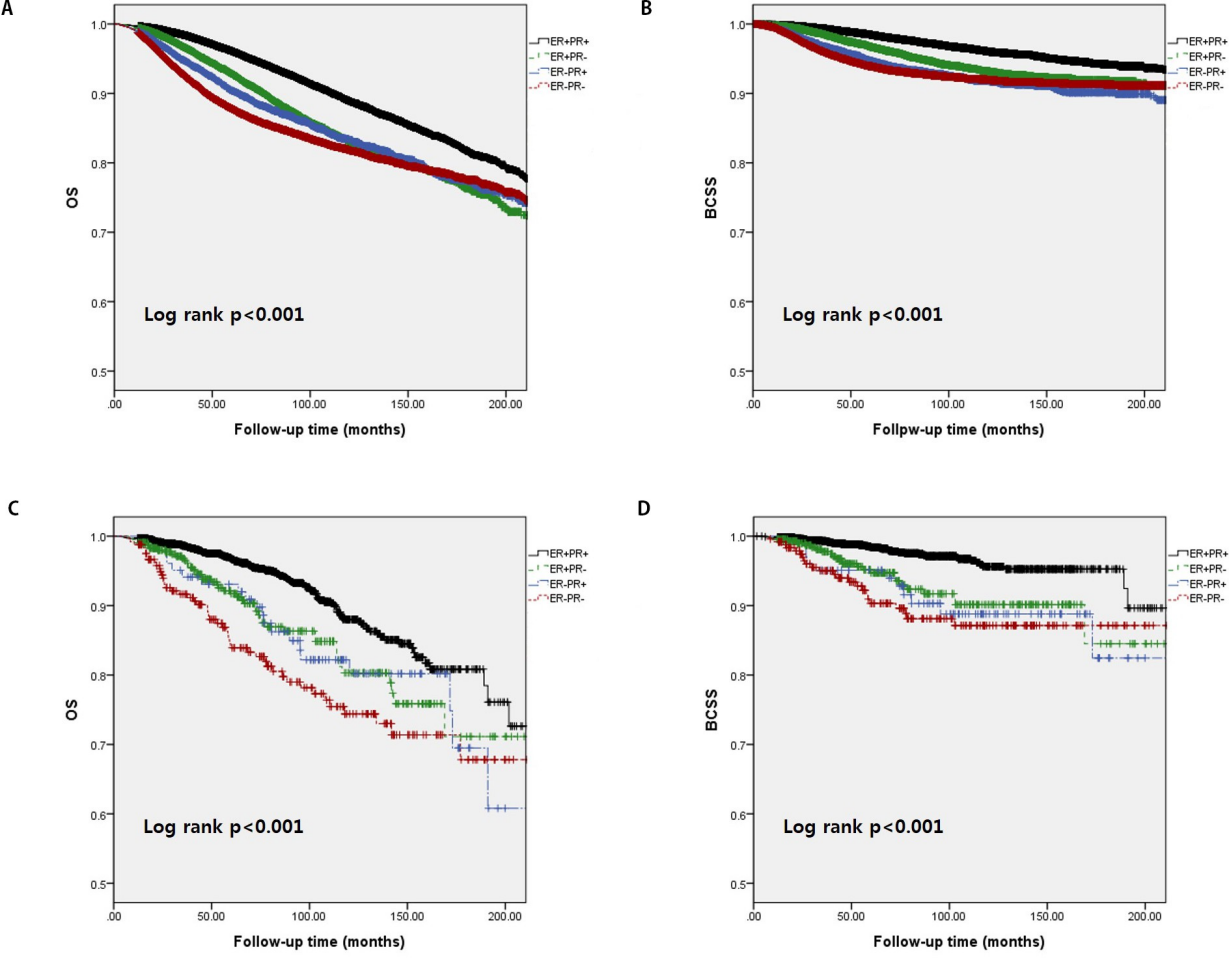
Kaplan–Meier survival analysis of overall survival (OS) (A, C) and breast cancer-specific survival (BCSS) (B, D) in the invasive ductal carcinoma (A, B) and invasive lobular carcinoma groups (C, D).

Similarly, as shown in Fig 3, the ER+/PR+ subgroup had the best survival, while the ER−/PR− subgroup had the worst in both the IDC and ILC groups (5-year OS in the IDC group: ER+/PR+, 88.9%; ER+/PR−, 83.0%; ER−/PR+, 83.4%; ER−/PR−, 81.9%; p < 0.001; 5-year BCSS of the IDC group: ER+/PR+, 96.1%; ER+/PR−, 93.2%; ER−/PR+, 91.8%; ER−/PR−, 92.0%; p < 0.001; 5-year OS of the ILC group: ER+/PR+, 88.0%; ER+/PR−, 80.3%; ER−/PR+, 82.2%; ER−/PR−, 74.4%; p < 0.001; 5-year BCSS of the ILC group: ER+/PR+, 95.6%; ER+/PR−, 90.1%; ER−/PR+, 88.8%; ER−/PR−, 87.1%; p < 0.001).

**Fig 4:**
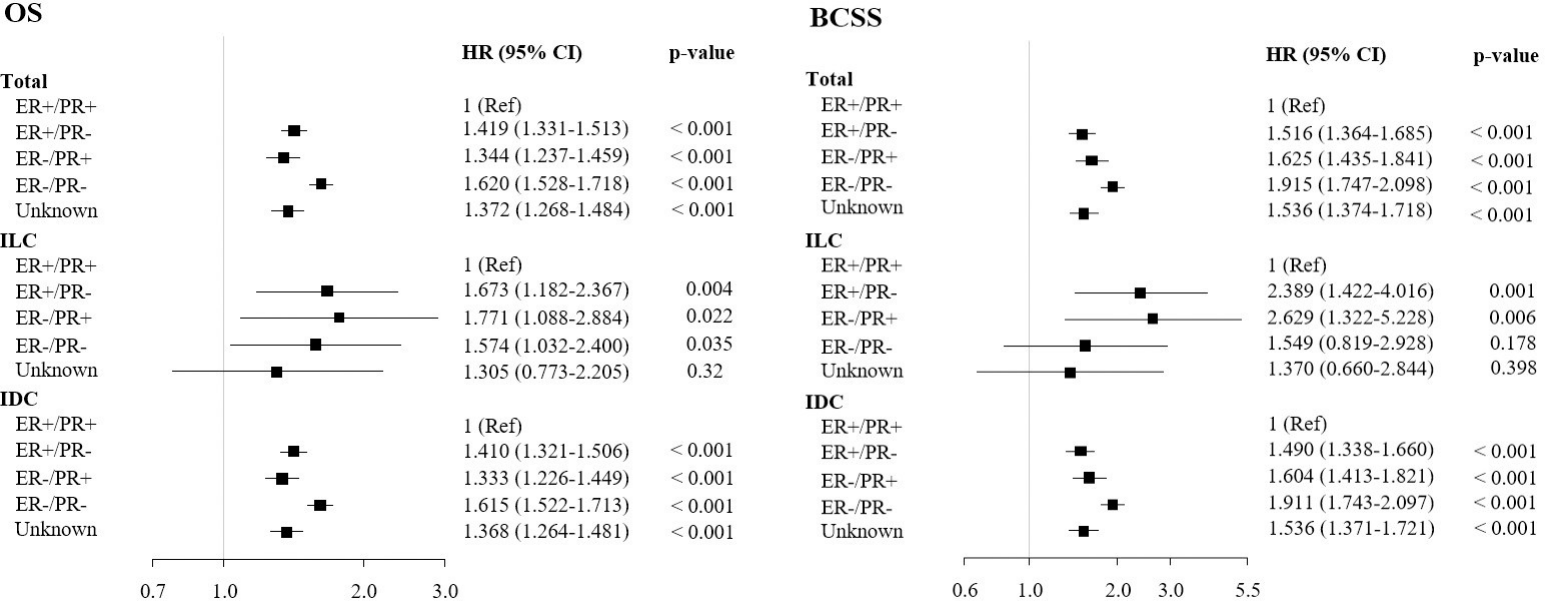
Cox regression analysis of survivals by hormone receptor expression.

The effects of hormone receptor expression status on survival outcomes are shown in Fig 4. In the total population, ER−/PR− status conferred the highest risk on OS (HR: 1.620, 95% CI: 1.528–1.718; p < 0.001), followed by ER+/PR− status (HR: 1.419, 95% CI: 1.331–1.513; p < 0.001), ER−/PR+ status (HR: 1.344, 95% CI: 1.237–1.459; p < 0.001), and ER−/PR− status (reference). Moreover, ER−/PR− status conferred the highest risk on BCSS among the total population (HR: 1.915, 95% CI: 1.747–2.098; p < 0.001), followed by ER−/PR+ status (HR: 1.625, 95% CI: 1.435–1.841; p < 0.001), ER+/PR− status (HR: 1.516, 95% CI: 1.364–1.685; p < 0.001), and ER+/PR+ status (reference). In the IDC group, the order of HR by hormone receptor expression was similar to that in the total population. In contrast, in the ILC group, ER−/PR+ status conferred the highest risk on OS (HR: 1.771, 95% CI: 1.088–2.884; p = 0.022), followed by ER+/PR− status (HR: 1.673, 95% CI: 1.182–2.367; p = 0.004), ER−/PR− status (HR: 1.574, 95% CI: 1.032–2.400; p = 0.035), and ER+/PR+ status (reference).

### Comparison of survival between IDC and ILC in each hormone receptor expression subgroup

We compared survival between the IDC and ILC populations in each hormone expression subgroup. There were no differences in survival between the IDC and ILC populations in the ER+/PR+ subgroup (5-year OS in IDC group: 88.9% vs. ILC group: 88.0%; p = 0.859; 5-year BCSS in IDC group 96.1% vs. ILC group: 95.6%; p = 0.828). There were similar results in the ER+/PR− subgroup (5-year OS in IDC group: 83.0 vs. ILC group: 80.3; p = 0.438; 5-year BCSS in IDC group: 93.2% vs. ILC group: 90.1%; p = 0.053) and the ER−/PR− subgroup (5-year OS in IDC group: 83.4% vs. ILC group: 82.2%; p = 0.522; 5-year BCSS in IDC group: 91.8% vs. ILC group: 88.8%; p = 0.291). However, the ER−/PR− subgroup showed differences in survival between the IDC and ILC populations (5-year OS in IDC group: 81.9% vs. ILC group: 74.4%; p = 0.040; 5-year BCSS in IDC group 92.0% vs. ILC group: 87.1%; p = 0.049). In the univariate Cox regression analysis, the HR of ILC in the ER−/PR− subgroup was 1.345 (95% CI: 1.012–1.788; p = 0.041).

## Discussion

In the present study, we compared the clinicopathological characteristics and survival outcomes of invasive breast cancer cases in Korea. In Asia, fewer studies than in the West have compared clinicopathological characteristics and survival between ILC and IDC cases of different molecular subtypes. According to a study using the SEER database, compared to patients with IDC, patients with ILC are older at diagnosis, have larger tumor size, show more advanced stage, have lower histological grade, and display more hormone expression positivity [6]. We found similar clinicopathological tendencies in the KBCR database. Several studies have asserted that ILC cases show larger tumor size and more advanced stage than IDC cases because the indistinct tumor growth pattern leads to delays in diagnosis and detection failure [16–19]. In the present study, total mastectomy was more common in patients with ILC than in those with IDC, as in other studies, probably because of the larger tumor size, more advanced stage, and multifocal tendency. For the comparison of survival between ILC and IDC cases, several studies have been carried out with conflicting results. In a study by Chen et al., ILC cases showed better OS before 60 months (HR of IDC vs. ILC: 1.118; p < 0.0001); thereafter, IDC cases showed better OS (HR of IDC vs. ILC: 0.775; p < 0.0001). The disease-specific survival curve showed that IDC cases had better survival outcomes than ILC cases did (HR of IDC vs. ILC: 0.809; p < 0.0001) [6]. In other reports, ILC cases had similar or better survival outcomes compared to those of IDC cases [8–14]. In the present study, there were no meaningful differences in OS or BCSS between patients with ILC and IDC in the KBCR database.

Breast cancer is a heterogeneous disease with varying hormone receptor status, and each subtype has different clinical features, treatment options, outcomes, and prognoses. For this reason, the hormone receptor subtypes are being studied worldwide. The ER+/PR+ subgroup presented the best survival in the present study, while the ER−/PR− subgroup presented the worst when the total study population was compared according to hormone expression status. The HR of ER+/PR− expression on OS was 1.419 (95% CI: 1.331–1.513; p < 0.001), while that of ER−/PR+ expression was 1.344 (95% CI: 1.237–1.459; p < 0.001) and that of ER −/PR− expression was 1.620 (95% CI: 1.528–1.718; p < 0.001) when ER+/PR+ expression was used as a reference. The HR of ER+/PR− expression on BCSS was 1.516 (95% CI: 1.364–1.685; p < 0.001), while that of ER−/PR+ expression was 1.625 (95% CI: 1.435–1.841; p < 0.001) and that of ER−/PR− expression was 1.915 (95% CI: 1.747–2.098; p < 0.001) when ER+/PR+ expression was used as a reference.

In the IDC group, the order of survival HR was similar to that in the total population. In contrast, the ILC group showed a slightly different order of survival HR. The HR of ER+/PR− expression on OS was 1.673 (95% CI: 1.182–2.367; p = 0.004), while that of ER−/PR+ expression was 1.771 (95% CI: 1.088–2.884; p < 0.022) and that of ER−/PR− expression was 1.574 (95% CI: 1.032–2.400; p < 0.035) when ER+/PR+ expression was used as a reference. The HR of ER+/PR− expression on BCSS was 2.389 (95% CI: 1.422–4.016; p = 0.001), while that of ER−/PR+ expression was 2.629 (95% CI: 1.322–5.228; p = 0.006) when ER+/PR+ expression was used as a reference.

**Fig 5:**
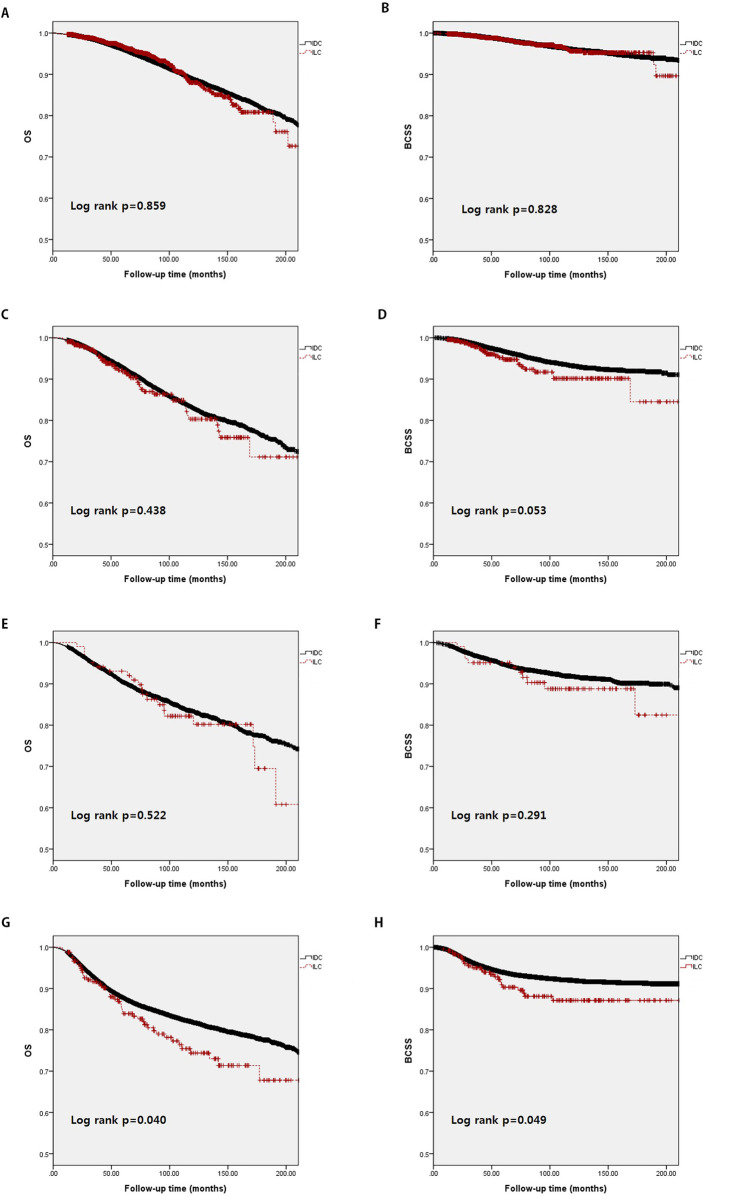
Kaplan–Meier survival analysis of overall survival (OS) (A, C, E, G) and breast cancer-specific survival (BCSS) (B, D, F, H), in the ER positive/PR positive (A, B), ER positive/PR negative (C, D), ER negative/PR positive (E, F), and ER negative/PR negative subgroups (G, H).

When analyzing each hormone receptor expression subgroup, as shown in Fig 5, there were no significant differences in survival between the IDC and ILC populations. However, the ER−/PR− subgroup showed differences in survival between the IDC and ILC populations (5-year OS in IDC group: 87.8% vs. ILC group: 87.5%; p = 0.040; 5-year BCSS in IDC group: 92.0% vs. ILC group: 87.1%; p = 0.049). In some studies, hormone receptor negativity was shown to reduce survival rates in the ILC group. In a study by Francesca et al., triple negative ILC showed the worst survival outcomes (79.7% at 5-years and 73.8% at 10-years) among all histological types. The same study reported that triple negative ILC showed a higher metastatic lymph node ratio (> 0.65) and lower response to chemotherapy than those of other triple negative breast cancer histological types [20]. It is hard to predict outcomes based on current classification and treatment regimens because the ER−/PR− population shows heterogeneity. Therefore, researchers must identify new molecular targets and subtypes. Understanding the heterogeneous molecular subtypes will allow targeted treatments in the future.

There were some limitations to this study. First, it was retrospective, so selection bias may have been present. However, the incidence of ILC is too small to study prospectively. Second, some data were clearly missing during the follow-up period, but we reasoned that the data were valuable and reliable because they were sourced from a large-scale database of one country with long-term follow-up and they corroborated findings of previous studies. Moreover, hormone receptor expression of 1–10% of tumor nuclei positivity was considered as negative, because it was initially registered as negative when the database started to be built. Another limitation may be that the neoadjuvant chemotherapy is not isolated, but in Korea, since it started in the 2010s, the number of patients who received neoadjuvant chemotherapy is small, so it will not have a significant effect.

In conclusion, we reviewed the clinicopathological characteristics and survival outcomes of invasive breast cancer cases in Korea. There was no difference in survival outcomes between ILC and IDC cases in the present study. However, in the ER−/PR− subgroup, the survival outcomes of ILC cases were worse than those of IDC cases. Given that the ER−/PR− group was heterogeneous and the incidence of ILC was low, further large studies are needed to allow comprehensive classification and identification of treatment regimens.

## Supporting information

S1 Table Inclusion/Exclusion criteria for patient selection. (DOCX)

S1 Data (XLSX)
