# Supplementary material for: Survival analysis in patients with invasive lobular cancer and invasive ductal cancer according to hormone receptor expression status in the Korean population
Source: PLoS One. 2022 Jan 20;17(1):e0262709. doi: 10.1371/journal.pone.0262709 (PMC8775332; doi:10.1371/journal.pone.0262709)
Supplement: S1 Table — (DOCX) [file pone.0262709.s001.docx]

**Supplementary Table 1.** Inclusion/Exclusion criteria for patient selection

| Inclusion criteria | Exclusion criteria |
| --- | --- |
| - All invasive breast cancer patients registered to the Korean Breast Cancer Registry (KBCR) database who aged more than 18 years old | - Patients with metastatic breast cancer at the time of diagnosis - Patients with carcinoma *in situ* - Patients with poorly evaluated axillary lymph nodes - Patients without biological subtype information |
